# The Surface Anatomy of the Abdominal Viscera

**Published:** 1901-08-17

**Authors:** Seymour Taylor

**Affiliations:** Physician at the West London Hospital


### HOSPITAL CLINICS AND MEDICAL PROGRESS.

### THE SURFACE ANATOMY OF THE ABDOMINAL VISCERA.

Being the abstract of a Lecture delivered in June, 1901, by SEYMOUR TAYLOR, M.D., F.R.C.P., Physician at the West London Hospital.

THE surface anatomy of the abdominal viscera is no less important, and probably more interesting, than even that of the thorax.

The situation of the umbilicus is an important landmark. A transverse section through it would about strike the top of the hip-bone, and would fall upon the fourth lumbar vertebra. It would also sever the abdominal aorta at its bifurcation. This is an important observation, inasmuch as aneurysmal dilatations of vessels which are given off from the aorta itself can only occur above this line, whilst aneurysms occurring below that line must arise in the common iliacs or their branches, always supposing that the sac of the aneurysm itself has not occurred in some of the terminal branches of the mesenteric vessels.

The skin on the belly presents important features. Pigmentation is seen in the line of the groins, and also in the middle line between the umbilicus and the pubes. Lineæ albicantes may sometimes be observed; even if they occur in the female they are no positive evidence of previous pregnancy although extremely suspicious of that; but they may arise from the collapse of the abdominal walls after an undue distension from whatever cause. Also the hair covering the pubes is differently arranged in the male as compared with the female. In the latter it is restricted entirely to the pubic eminence, whereas in the adult male it is pyramidal in shape, extends up to the umbilicus, and may even be continuous with the hairy covering of the front of the chest.

There are one or two well-marked lines in the abdominal wall to which it is necessary to draw attention. That in the middle line known as the linea alba is perhaps the most important from the surgical point of view, inasmuch as the surgeon in the great majority of instances cuts through this line to open the abdominal cavity, as he thereby avoids any muscles and the consequent hæmorrhage that would arise if muscular tissue were cut. The linea semilunaris is outside this and marks the outer boundary of the rectus muscle. It practically runs from the eighth costal cartilage down to the spine of the pubes, but in a curved line, the concavity being inwards and with its widest part opposite the umbilicus. There are other transverse lines marking the septa in the rectus muscle, but they would appear to be of no great surgical or medical importance except that one of them between the umbilicus and the xiphoid may be mistaken for the lower line of the liver.

The abdominal walls as a whole bear very strong resemblance to those of a stomach or urinary bladder or of other hollow viscus, for they consist of a muscular coat comparable to the involuntary muscular fibres of, say, the bladder: beneath this is a strong fibrous tunic and yet again comes the serous coat. Now of all these three tunics which compose the abdominal wall, the fascia transversalis is perhaps the most interesting. It is not so strong in man as in the lower animals, since its principal function, namely that of holding up the intestines, is not so severely taxed as it is in quadrupeds in which the intestines are pendulous. Nevertheless, it is a strong membrane, and is thickest where it would be expected to be thickest, namely in the lower regions of the abdomen where most pressure of the intestines would be exerted. Merging behind with the transversalis muscle, it may be traced backwards to the back-bone, where, it splits into three septa, one enveloping the psoas, another going to the transverse processes of the lumbar vertebræ, whilst the third, encasing the lumbar muscles, is attached to the spines of the vertebræ. This third lamina is important in the painful affection known as lumbago for the inflammatory swelling of the great group of muscles known as the erector spinæ is limited and confined by the sheathing of this fascia. You will thus see what speedy relief can be frequently obtained for a sufferer from lumbago by puncture of the erector spinæ muscles through this posterior lamina or fascia.

The groin will next occupy our attention for a few minutes. It is the fold between the thigh and the belly. This fold is occupied by Poupart's ligament. The glands of the groin are of two sets: those which run horizontal to Poupart's ligament and receive the lymphatics from the scrotum, whilst the lymphatics from the lower limb are for the most part drained into those glands which occupy the upper part of the thigh and run vertically. With this recollection it will not do to condemn a patient as having some inoculation, venereal or otherwise, of scrotum or penis unless you can satisfy yourself that the genital lymphatics are involved. I may here remind you [illegible]t the lymphatics of the testicle itself drain into those [illegible]ep glands of the loin near the kidneys, so that any specific or malignant affection of the testis would produce secondary glandular enlargement, not of the groin but of the deep parts of the abdomen, for you will recollect that the testis itself is an emigrant which, having left the locality of its birth travels through the abdominal rings into the scrotum; but it keeps up its connection with the loin by means of its lymphatic vessels. Perhaps the most important bony point in this region is the spine of the pubes, which can be easily felt, no matter how stout the subject may be. This spine of the pubes is situated at the outer end of the pubic crest which runs outwards from the symphysis. These two bony points are important, inasmuch as the crest itself forms the base of that triangle known as the external abdominal ring, whilst the outer portion of the same ring is attached to the pubic spine, and the inner portion interlaces with its fellow of the opposite side above the symphysis. With these anatomical points in your mind it is obvious that any hernia escaping from the abdomen through the external abdominal ring, and therefore internal to the spine of the pubes, must be an inguinal hernia, whilst any hernia which emerges from the abdomen external to the spine of the pubes must pass below Poupart's ligament into the thigh and is therefore a femoral hernia. Hesselbach's triangle is an important surgical space concerned in the operation for inguinal hernia. It is a small triangle, the inner boundary of which is the linea semilunaris or outer edge of the rectus; the external boundary is the deep epigastric artery, whilst the base of the triangle is formed by Poupart's ligament. Now the deep epigastric artery runs from the external iliac about the middle of Poupart's ligament upwards and inwards in a direction towards but below the umbilicus. This again is an important anatomical landmark, inasmuch as it has the spermatic cord in front of, external to, and behind it; it also marks the inner margin of the internal abdominal ring.

(*To be continued.*)

